# Sleep and liver function biomarkers in relation to risk of incident liver cancer: a nationwide prospective cohort study

**DOI:** 10.1186/s12916-024-03440-w

**Published:** 2024-06-24

**Authors:** Jiahao Song, Lieyang Fan, Da Shi, Xuefeng Lai, Hao Wang, Wei Liu, Linling Yu, Ruyi Liang, Yongfang Zhang, Shuhui Wan, Yueru Yang, Bin Wang

**Affiliations:** 1 Department of Occupational and Environmental Health, School of Public Health, Tongji Medical College, Huazhong University of Science and Technology, Wuhan, Hubei 430030 China; 2 Key Laboratory of Environment and Health, Ministry of Education & Ministry of Environmental Protection, and State Key Laboratory of Environmental Health (Incubating), School of Public Health, Tongji Medical College, Huazhong University of Science and Technology, Wuhan, Hubei 430030 China; 3 Agricultural, Food and Nutritional Science, Faculty of Agricultural, Life and Environmental Sciences, University of Alberta, Edmonton, T6G 2P5 AB Canada; 4 Department of Epidemiology and Biostatistics, School of Public Health, Tongji Medical College, Huazhong University of Science and Technology, Wuhan, Hubei 430030 China

**Keywords:** Liver cancer, Sleep, Liver function biomarker, Joint association, Prospective cohort study

## Abstract

**Background:**

To assess the largely undetermined separate and joint effects of sleep and liver function biomarkers on liver cancer.

**Methods:**

Data of 356,894 participants without cancer at baseline in the UK Biobank were analyzed. Sleep score was evaluated using five sleep traits (sleep duration, chronotype, insomnia, snoring, and excessive daytime sleepiness) and dichotomized into healthy or unhealthy sleep. Circulating liver function biomarkers were measured. Cox proportional hazard model was performed to investigate the independent and joint associations of sleep and liver function biomarkers with liver cancer incidence.

**Results:**

After a median follow-up time of 13.1 years, 394 cases of incident liver cancer were documented. The multivariable-adjusted hazard ratio (HR) for liver cancer was 1.46 (95% confidence interval: 1.15–1.85) associated with unhealthy sleep (vs. healthy sleep), and was 1.17 (1.15–1.20), 1.20 (1.18–1.22), 1.69 (1.47–1.93), 1.06 (1.06–1.07), 1.08 (1.07–1.09), 1.81 (1.37–2.39), or 0.29 (0.18–0.46) associated with each 10-unit increase in alanine transaminase (ALT), aspartate transaminase (AST), total bilirubin (TBIL), gamma-glutamyl transferase (GGT), alkaline phosphatase (ALP), total protein (TP), or albumin (ALB), respectively. Individuals with unhealthy sleep and high (≥ median) ALT, AST, TBIL, GGT, ALP, or TP or low (< median) ALB level had the highest HR of 3.65 (2.43–5.48), 4.03 (2.69–6.03), 1.97 (1.40–2.77), 4.69 (2.98–7.37), 2.51 (1.75–3.59), 2.09 (1.51–2.89), or 2.22 (1.55–3.17) for liver cancer, respectively. Significant additive interaction of unhealthy sleep with high TP level on liver cancer was observed with relative excess risk due to an interaction of 0.80 (0.19–1.41).

**Conclusions:**

Unhealthy sleep was associated with an increased risk of liver cancer, especially in participants with lower ALB levels or higher levels of ALT, AST, TBIL, GGT, ALP, or particularly TP.

**Supplementary Information:**

The online version contains supplementary material available at 10.1186/s12916-024-03440-w.

## Background

Liver cancer ranks as the seventh most commonly diagnosed cancer worldwide and the third leading cause of cancer-related death [1]. In 2020, there were 905,700 new cases and 830,200 deaths from liver cancer globally. Furthermore, the incidence and mortality of liver cancer are predicted to increase by over 55% by 2040 [2]. Unhealthy lifestyle factors, such as weight gain, cigarette smoking, alcohol consumption, physical inactivity, and unhealthy dietary patterns, have been associated with increased liver cancer risk [3–6]. Sleep is a crucial aspect of lifestyle and a healthy sleep is essential for physical and mental health [7]. Unhealthy sleep patterns, such as unfavorable sleep duration, snoring, and insomnia, have been associated with increased morbidity and mortality from lung, colorectal, and all-caused cancer [8–13]. For instance, a prospective study of 469,691 individuals in the UK Biobank indicated that unfavorable sleep duration and evening chronotype were associated with an increased risk of lung cancer [8]. In addition, Song et al. investigated the association between sleep quality and incident all-caused cancer risk based on 10,036 participants from an elderly cohort and found that poor sleep quality was positively associated with the long-term risk of developing cancer [10]. Although these studies provided considerable insight that unhealthy sleep could increase the risk of cancer, evidence concerning the liver cancer risk from sleep remains absent.

Liver function biomarkers, such as alanine transaminase (ALT), aspartate transaminase (AST), gamma-glutamyl transferase (GGT), albumin (ALB), alkaline phosphatase (ALP), total bilirubin (TBIL), and total protein (TP), are critical indicators of liver biosynthesis, metabolism, detoxification, as well as overall nutritional and immune status [14]. These markers are usually included in routine blood tests and are widely used to assess liver health and liver cancer risk [15, 16]. Based on 235 hepatocellular carcinoma (HCC) patients and 259 healthy controls with negative alpha-fetoprotein, Li and colleagues found that GGT/ALP combined with GGT/ALT and ALT/AST were effective diagnostic markers of alpha-fetoprotein-negative HCC, especially in patients with normal liver function or early stage [17]. Another clinical study of 414 HCC patients in Anhui, China, aimed to evaluate the prognostic significance of serum AST/ALT and GGT levels revealed that ALT, AST, and GGT were potential indicators of liver cancer progression [18]. Nevertheless, these studies were somewhat restricted by small-sized/unrepresentative samples or ultimate liver cancer mortality outcomes. Therefore, it is essential to investigate the relationships between the liver function biomarkers and the risk of incident liver cancer in the general population with a large sample size.

In the current study, we conducted systematic analyses based on the UK Biobank, a nationwide large-scale general population-based prospective cohort, to investigate the relationships of sleep traits and comprehensive sleep score with the risk of incident liver cancer. Potential associations between liver function biomarkers and the risk of incident liver cancer were also evaluated. Furthermore, we examined the joint associations of sleep and liver function biomarkers with the risk of incident liver cancer and assessed the potential additive interactions.

## Methods

### Study participants

The UK Biobank is a prospective cohort with over 500,000 participants of adults aged 37 ~ 73 years old recruited from 22 centers across the UK between 2006 and 2010 (https://www.ukbiobank.ac.uk/media/gnkeyh2q/study-rationale.pdf). Participants completed questionnaires, interviews, and physical measurements at baseline assessments. Blood samples were collected from all participants at recruitment for detecting circulating biomarkers. All participants consented to the use of their de-identified data and access to their national health-related hospital and death records. Detailed study design and methods of the cohort were described elsewhere [19].

**Fig. 1:**
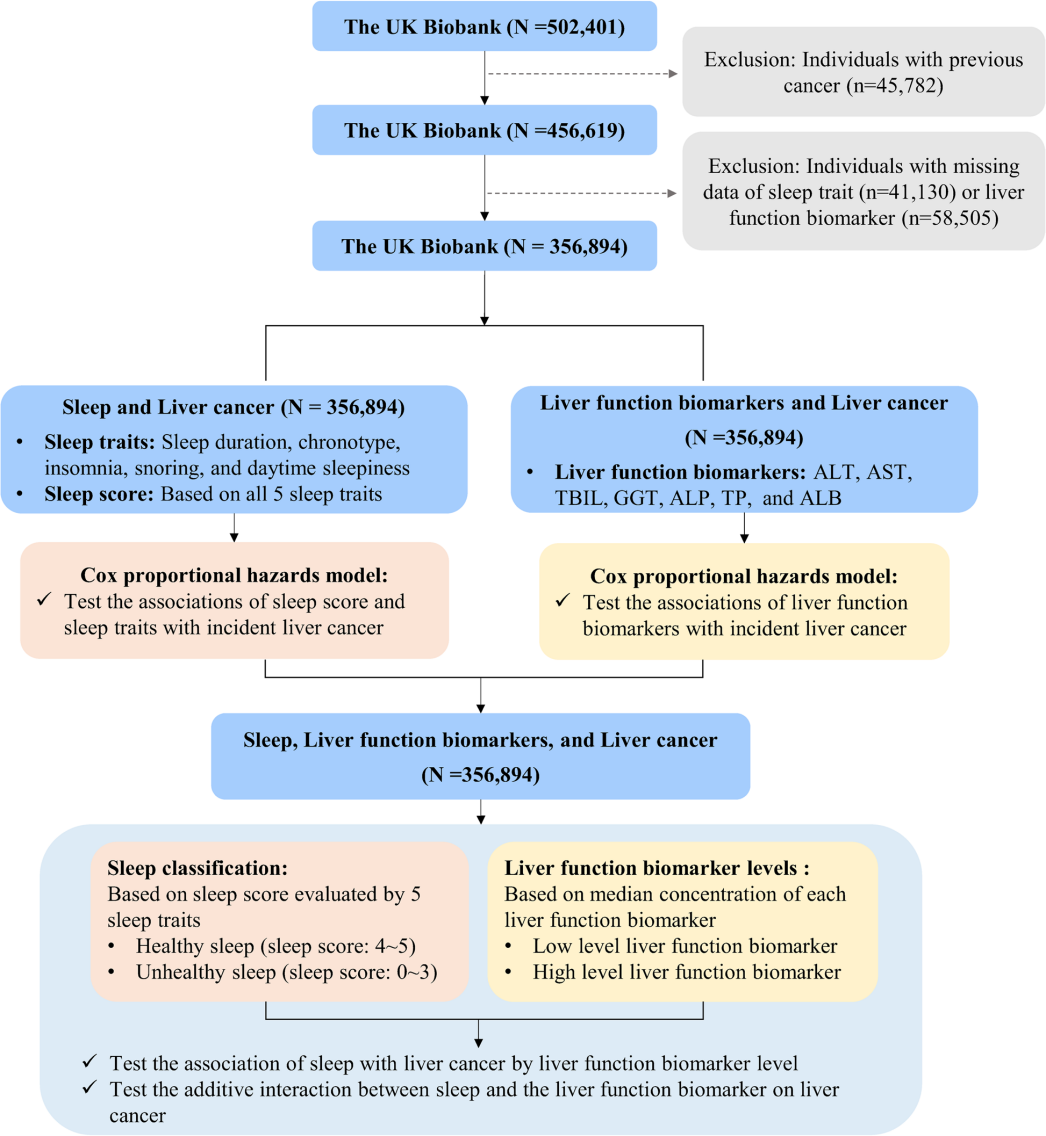
Flow diagram of the study design and workflow. Abbreviations: ALT, alanine transaminase; AST, aspartate transaminase; TBIL, total bilirubin; GGT, gamma-glutamyl transferase; ALP, alkaline phosphatase; TP, total protein; ALB, albumin

We excluded participants with prevalent cancer at recruitment (*n* = 45,782); missing data on any of the five sleep traits (*n* = 41,130); or missing data on any of the seven liver function biomarkers (*n* = 58,505). Eventually, a total of 356,894 participants were included in our analysis. A detailed study design and workflow of this study is displayed in Fig. 1.

### Definition of sleep trait and sleep score

All sleep traits were collected through a touchscreen questionnaire. A sleep score was created by combining five independent sleep traits including sleep duration, morning/evening chronotype, insomnia, snoring, and excessive daytime sleepiness [20, 21]. Online supplemental Additional file 1: Table S1 provides a detailed questionnaire and definition of each item. Healthy sleep traits were defined as favorable sleep duration (sleep 7 ~ 8 h/day); morning chronotype (“morning” person or more a “morning” than an “evening” person); no usual insomnia (“never/rarely” or “sometimes”); no snoring; and no dozing (“never/rarely” or “sometimes”). Each of the independent sleep traits was coded 1 if meeting the healthy criterion and 0 if not. The sleep score was obtained by summing up the 5 independent sleep traits, and all participants were subsequently categorized into two sleep groups accordingly: “healthy sleep” (sleep score ≥ 4) and “unhealthy sleep” (sleep score 0 ~ 3) [20–22].

### Blood collection and laboratory methods

Blood samples were collected from all participants at recruitment, then labeled, centrifuged, and stored at – 80 ℃. Circulating levels of ALT, AST, GGT, and ALP were determined using the enzymatic rate method (Beckman Coulter AU5800); TBIL, TP, and ALB were determined using the colorimetric method; and high sensitivity C-reactive protein (CRP) was determined using the immuno-turbidimetric method. A detailed introduction of the assay performance has been published elsewhere [23]. The average within-laboratory coefficients of variation in quality-control samples for ALT, AST, TBIL, GGT, ALP, TP, ALB, and CRP ranged from 1.16 ~ 2.91%, 1.33 ~ 2.13%, 1.48 ~ 1.92%, 1.44 ~ 2.84%, 2.84 ~ 3.08%, 1.09 ~ 1.22%, 2.09 ~ 2.20%, and 1.69 ~ 2.31%, respectively [23].

According to the published literature, the reproducibilities of all liver biomarkers in the subsample of participants with repeat measurements were fair to good (intraclass correlation coefficients: 0.40 ~ 0.74) or excellent (intraclass correlation coefficients: ≥ 0.75) in the UK Biobank [16, 24]. Therefore, the concentration of liver function biomarkers at baseline were justifiably used for analysis in this study in accordance with previous studies [25, 26].

### Assessment of outcome

Incidence of liver cancer was collected until July 19, 2022. The identification of cancer cases in the UK Biobank cohort was carried out by connecting with the national cancer registries of England, Wales, and Scotland. All diseases were confirmed according to the 10th Revision of the International Classification of Diseases (ICD-10), and liver cancer was defined as ICD-10 code C22 [2]. Participants were followed up from the enrollment until the time of liver cancer diagnosis or censoring. Censoring was defined as the time of death, withdrawal from the study, or the end of follow-up, whichever came first.

### Covariates

To reduce the effect of potential confounding, demographics and contextual covariates were selected based on published literature and statistic consideration [6, 22, 27, 28], including age at recruitment (continuous), sex (male/female), ethnicity (White/others), body mass index (BMI; continuous), smoking status (never/former/current), alcohol consumption (never or seldom/1 ~ 4 times per week/almost daily), healthy diet (yes/no; healthy diet takes 10 diet components [fruits, vegetables, fish, dairy, vegetable oils, processed meats, unprocessed meats, whole grains, refined grains, and sugar-sweetened beverages] into account) [29], physical activity (low/moderate/high) [30], any treatment/medication taken (never/ever), Townsend deprivation index (indicative of socioeconomic status; continuous), family history of cancer (yes/no), and CRP (continuous).

### Statistical analysis

Descriptive statistics were presented and stratified by the participants with or without incident liver cancer. *T*-test/chi-squared test was used to compare the differences in demographic characteristics and levels of liver function biomarkers between participants with and without incident liver cancer for continuous/categorical variables. Spearman’s correlation analysis was used to estimate the correlation between liver function biomarkers.

Cox proportional hazard model was performed to assess the hazard ratio (HR) and 95% confidence interval (95% CI) for the risk of incident liver cancer associated with sleep score and independent sleep traits, whereas the model 1 was a crude model; the model 2 was adjusted for age (continuous), sex (male/female), ethnicity (White/others), BMI (continuous), smoking status (never/former/current), alcohol consumption (never or seldom/1 ~ 4 times per week/almost daily), healthy diet (yes/no), and physical activity (low/moderate/high); and the model 3 was further adjusted for any treatment/medication taken (never/ever), Townsend deprivation index (continuous), family history of cancer (yes/no), and CRP (continuous) based on model 2. The Kaplan–Meier curves of the cumulative risks and event rates were also presented along with the corresponding HR and 95% CI. We further investigated the association of sleep duration with the risk of incident liver cancer across different sleep durations with the favorable sleep duration (7 ~ 8 h) serving as a reference group [8, 20].

Associations of liver function biomarkers with the risk of liver cancer incidence were estimated by Cox proportional hazard models with adjustment for confounders mentioned above. We also estimated the HR (95% CI) of liver cancer incidence risk for the second (25th ~ 50th percentile), third (50th ~ 75th percentile), and fourth (the highest; ≥ 75th percentile) quartiles of these liver function biomarkers compared to the first (the lowest; < 25th percentile) quartile. Restricted cubic spline regression was used to investigate the dose–response relationships between liver function biomarkers and the risk of incident liver cancer. In addition, stratified analyses were conducted according to several major characteristics, including age at recruitment (< 60/ ≥ 60 years), sex (male/female), ethnicity (White/others), BMI (< 25/ ≥ 25 kg/m^2^), smoking status (never/former/current), and alcohol consumption (never or seldom/1 ~ 4 times per week/almost daily). The modification effect was estimated by adding a product term of liver function biomarker and stratified variable into the statistic model.

To examine the robustness of the associations between sleep or liver function biomarkers and incident liver cancer, we performed sensitivity analysis by (1) excluding incident liver cancer in the first 2 years of follow-up (to minimize the possibility of reverse causality); (2) excluding individuals with liver disease at baseline; (3) additionally adjusting for the liver function biomarkers, which were included as categorical variables rather than continuous variables to avoid collinearity, when assessing the association between sleep and incident liver cancer; (4) additionally adjusting for sleep score (healthy/unhealthy) and the other liver function biomarkers when assessing the association between one liver function biomarker and incident liver cancer; (5) performing inverse probability weighting (IPW) analysis to account for missing data and avoid potential selection bias; and (6) conducting competing risk model to account for mortality.

To investigate the joint associations of sleep and liver function biomarkers with liver cancer incidence risk, all participants were divided into four groups based on sleep score (healthy sleep: sleep score ≥ 4; unhealthy sleep: sleep score 0 ~ 3) and liver function biomarker levels (high: ≥ median concentration; low: < median concentration). We evaluated the HR (95% CI) of liver cancer incidence risk in the other three groups, using participants with healthy sleep and whose liver function biomarker levels tended to be at a lower risk of liver cancer incidence as the reference group. The additive interaction was assessed by using two indexes: relative excess risk due to interaction (RERI) and attributable proportion due to interaction (AP), and the 95% CIs of RERI and AP include 0 indicating no additive interaction [31].

Schoenfeld residuals were used to test the proportional hazard assumption and no violation was found. All analyses were performed in R software, version 4.2.2 (R Foundation for Statistical Computing). All statistical tests were two-sided, and *P* < 0.05 was considered to indicate statistical significance. Cox regression was performed using the R package *survival*, and additive interaction was estimated using the R package *epiR*.

## Results

### Characteristics of study participants

Demographic characteristics of participants included in the study are displayed in Table 1. After a median follow-up time of 13.1 years, 394 cases of incident liver cancer (168 in men and 280 in women) were documented. Compared with participants without incident liver cancer, those with incident liver cancer had higher BMI levels, higher CRP levels, worse socioeconomic status, and less physical activity and were older, more likely to consume alcohol, and less likely to have never smoked or never taken any treatment/medication. Additionally, the proportion of the population with unhealthy sleep scores, unfavorable sleep duration, or insomnia was higher in participants with incident liver cancer compared to participants without incident liver cancer. Moreover, all liver function biomarkers at baseline were higher in incident liver cancer individuals except that ALB was lower. These liver function biomarkers were significantly correlated with each other (all *P* < 0.001) (Table 2).

**Table 1:** Demographic characteristics at baseline of participants included in the study (*N* = 356,894)

Characteristics	Overall (*N* = 356,894)	Without incident liver cancer (*N* = 356,500)	Incident liver cancer (*N* = 394)	*P* value^c^
Age, year, mean ± SD	56.20 ± 8.10	56.19 ± 8.10	61.04 ± 6.22	** < 0.001**
Male, *N* (%)	170,831 (47.87%)	1705,51 (47.84%)	280 (71.07%)	** < 0.001**
BMI, kg/m^2^, mean ± SD	27.40 ± 4.73	27.40 ± 4.73	29.29 ± 5.24	** < 0.001**
Townsend deprivation index^a^, mean ± SD	− 1.40 ± 3.04	− 1.40 ± 3.04	− 0.95 ± 3.19	**0.005**
Ethnicity, White, *N* (%)	324,246 (90.85%)	3238,86 (90.85%)	360 (91.37%)	0.700
Family history of cancer, *N* (%)	71,442 (20.02%)	71,351 (20.01%)	91 (23.1%)	0.100
CRP, mg/L, mean ± SD	2.53 ± 4.21	2.53 ± 4.21	3.81 ± 5.40	** < 0.001**
Healthy diet^b^,* N* (%)	194,268 (54.43%)	194,057 (54.43%)	211 (53.55%)	0.760
Smoking status, *N* (%)				** < 0.001**
Never	195,933 (54.90%)	195,796 (54.92%)	137 (34.77%)	
Former	123,072 (34.48%)	122,875 (34.47%)	197 (50.00%)	
Current	36,793 (10.31%)	36,734 (10.3%)	59 (14.97%)	
Unknown	1096 (0.31%)	1095 (0.31%)	1 (0.25%)	
Alcohol consumption, *N* (%)				**0.002**
Never/Seldom	104,756 (29.35%)	104,621 (29.35%)	135 (34.26%)	
1 ~ 4 times per week	177,622 (49.77%)	177,461 (49.78%)	161 (40.86%)	
Almost daily	74,298 (20.82%)	74,200 (20.81%)	98 (24.87%)	
Unknown	218 (0.06%)	218 (0.06%)	0 (0%)	
Any treatment/medication taken, *N* (%)				** < 0.001**
Never	101,497 (28.44%)	101,429 (28.45%)	68 (17.26%)	
Ever	255,319 (71.54%)	254,993 (71.53%)	326 (82.74%)	
Unknown	78 (0.02%)	78 (0.02%)	0 (0%)	
Physical activity, *N* (%)				**0.006**
Low	54,757 (15.34%)	54,677 (15.34%)	80 (20.30%)	
Moderate	119,098 (33.37%)	118,977 (33.37%)	121 (30.71%)	
High	119,246 (33.41%)	119,134 (33.42%)	112 (28.43%)	
Unknown	63,793 (17.87%)	63,712 (17.87%)	81 (20.56%)	
Independent sleep traits, *N* (%)				
Unfavorable sleep duration	113,291 (31.74%)	113,131 (31.73%)	160 (40.61%)	** < 0.001**
Evening chronotype	62,615 (17.54%)	62,541 (17.54%)	74 (18.78%)	0.600
Insomnia	96,981 (27.17%)	96,840 (27.16%)	141 (35.79%)	** < 0.001**
Snoring	222,409 (62.32%)	222,178 (62.32%)	231 (58.63%)	0.100
Excessive daytime sleepiness	9604 (2.69%)	9588 (2.69%)	16 (4.06%)	0.100
Sleep score, *N* (%)				**0.004**
Healthy (4 ~ 5)	209,976 (58.83%)	209,773 (58.84%)	203 (51.52%)	
Unhealthy (0 ~ 3)	146,918 (41.17%)	146,727 (41.16%)	191 (48.48%)	

*Abbreviations*: *BMI* body mass index, *CRP* C-reaction protein, *SD* standard deviation

^a^Positive value of the index will indicate areas with high material deprivation, whereas those with a negative value will indicate relative affluence

^b^Healthy diet takes 10 diet components including fruits, vegetables, fish, dairy, vegetable oils, processed meats, unprocessed meats, whole grains, refined grains, and sugar-sweetened beverages into account. Adequate intake of at least half of all diet components was considered as a healthy diet, and less than half was considered an unhealthy diet

^c^*T*-test/chi-squared test was used to compare the differences in basic characteristics between participants without incident liver cancer and participants with incident liver cancer for continuous/categorical variables

**Table 2:** Basic statistics of liver function biomarkers and correlation matrix (*N* = 356,894)

Liver function biomarkers, mean ± SD	Overall (*N* = 356,894)	Without incident liver cancer (*N* = 356,500)	Incident liver cancer (*N* = 394)	*P* value^a^	Spearman correlation coefficient^b^						
					ALT	AST	TBIL	GGT	ALP	TP	ALB
ALT (U/L)	23.64 ± 14.13	23.62 ± 14.08	40.56 ± 32.68	** < 0.001**	1	0.70	0.12	0.60	0.18	0.13	0.14
AST (U/L)	26.19 ± 10.04	26.17 ± 9.98	43.88 ± 31.08	** < 0.001**	-	1	0.16	0.43	0.15	0.18	0.15
TBIL (μmol//L)	9.19 ± 4.43	9.19 ± 4.43	11.16 ± 6.50	** < 0.001**	-	-	1	0.10	− 0.11	0.08	0.21
GGT (U/L)	37.40 ± 41.06	37.29 ± 40.50	136.62 ± 182.25	** < 0.001**	-	-	-	1	0.23	0.15	0.11
ALP (U/L)	82.89 ± 25.10	82.87 ± 24.97	106.67 ± 76.01	** < 0.001**	-	-	-	-	1	0.11	− 0.03
TP (g/L)	72.54 ± 4.08	72.54 ± 4.08	73.40 ± 4.80	** < 0.001**	-	-	-	-	-	1	0.48
ALB (g/L)	45.26 ± 2.61	45.26 ± 2.61	44.12 ± 3.12	** < 0.001**	-	-	-	-	-	-	1

*Abbreviations*: *ALT* alanine transaminase, *AST* aspartate transaminase, *TBIL* total bilirubin, *GGT* gamma-glutamyl transferase, *ALP* alkaline phosphatase, *TP* total protein, *ALB* albumin

^a^*T*-test test was used to compare the differences in liver function biomarkers between participants without incident liver cancer and participants with incident liver cancer

^b^All *P* < 0.001

### Association between sleep and liver cancer incidence

Table 3 presents the associations of sleep traits and sleep score with liver cancer incidence risk. Across all three models, an unhealthy sleep (vs. healthy sleep) was found to be associated with an increased risk of liver cancer incidence. The HR (95% CI) for liver cancer incidence was 1.35 (1.11, 1.64) for individuals with unhealthy sleep compared to those with healthy sleep (Model 1). The association did not appreciably change with adjustment for age, sex, ethnicity, BMI, smoking status, alcohol consumption, healthy diet, and physical activity (Model 2; HR = 1.43, 95% CI: 1.14, 1.79), and with additional adjustment for any treatment/medication taken, Townsend deprivation index, family history of cancer, and C-reaction protein (Model 3; HR = 1.46, 95% CI: 1.15, 1.85). Moreover, for a per 1-unit increase in sleep score, the multivariate-adjusted HR (95% CI) for incident liver cancer was 1.24 (1.11, 1.39) (Additional file 1: Table S2). For independent sleep trait, in the multivariable-adjusted model (Model 3), unfavorable sleep duration, insomnia, and snoring were each independently associated with 29% (HR = 1.29, 95% CI: 1.01, 1.65), 43% (HR = 1.43, 95% CI: 1.11, 1.83), and 30% (HR = 1.30, 95% CI: 1.01, 1.67) increased risk of liver cancer incidence, respectively. Kaplan–Meier curves generally revealed similar results (Additional file 1: Fig. S1). Furthermore, compared with the favorable sleep duration (7 ~ 8 h), sleep duration < 6 h or > 9 h was associated with 95% (Model 3; HR = 1.95, 95% CI: 1.30, 2.93) or 77% (Model 3; HR = 1.77, 95% CI: 0.93, 3.38) increased risk of liver cancer incidence, respectively, revealing a U-shaped association between sleep duration and risk of liver cancer incidence (Additional file 1: Fig. S2).

**Table 3:** Association of sleep with the risk of incident liver cancer (*N* = 356,894)

Sleep	Model 1	Model 2	Model 3
	HR (95% CI)	HR (95% CI)	HR (95% CI)
Unhealthy sleep (sleep score: 0 ~ 3)	**1.35 (1.11, 1.64)**	**1.43 (1.14, 1.79)**	**1.46 (1.15, 1.85)**
Independent sleep trait			
Unfavorable sleep duration	**1.48 (1.21, 1.81)**	**1.30 (1.03, 1.63)**	**1.29 (1.01, 1.65)**
Evening chronotype	1.07 (0.83, 1.38)	1.28 (0.95, 1.73)	1.18 (0.85, 1.64)
Insomnia	**1.51 (1.23, 1.86)**	**1.37 (1.08, 1.73)**	**1.43 (1.11, 1.83)**
Snoring	0.85 (0.70, 1.04)	1.24 (0.98, 1.56)	**1.30 (1.01, 1.67)**
Excessive daytime sleepiness	1.57 (0.95, 2.59)	1.17 (0.67, 2.05)	1.28 (0.73, 2.25)

Model 1 was a crude model; Model 2 was adjusted for age, sex, ethnicity, BMI, smoking status, alcohol consumption, healthy diet, and physical activity; Model 3 was adjusted for age, sex, ethnicity, BMI, smoking status, alcohol consumption, healthy diet, physical activity, any treatment/medication taken, Townsend deprivation index, family history of cancer, and CRP

*Abbreviations*: *HR* hazard ratio, *CI* confidence interval

### Associations between liver function biomarkers and liver cancer incidence

Circulating levels of liver function biomarkers were significantly and linearly (all *P* total < 0.001 and all *P* nonlinear > 0.1; Additional file 1: Fig. S3) associated with liver cancer incidence in dose–response manners (all *P* and *P* trend < 0.05) (Table 4). Compared with individuals with the lowest quartile (Q1) of liver function biomarkers, the HRs (95% CIs) of those in the highest quartile (Q4) were 3.25 (2.18, 4.84) for ALT, 3.55 (2.45, 5.15) for AST, 1.68 (1.18, 2.38) for TBIL, 5.17 (3.22, 8.32) for GGT, 2.12 (1.49, 3.00) for ALP, 2.13 (1.51, 3.01) for TP, and 0.66 (0.47, 0.94) for ALB. In the multivariable model with continuous liver function biomarkers, for each 10-unit increase in ALT, AST, TBIL, GGT, ALP, TP, or ALB, the risk of liver cancer incidence increased by 17% (HR = 1.17, 95% CI: 1.15, 1.20), 20% (HR = 1.20, 95% CI: 1.18, 1.22), 69% (HR = 1.69, 95% CI: 1.47, 1.93), 6% (HR = 1.06, 95% CI: 1.06, 1.07), 8% (HR = 1.08, 95% CI: 1.07, 1.09), 82% (HR = 1.81, 95% CI: 1.37, 2.39), or decreased by 71% (HR = 0.29, 95% CI: 0.18, 0.46), respectively. Further stratified analysis showed that sex, BMI, smoking status, and alcohol consumption significantly modified the relationships of ALB, AST, ALT, and GGT with liver cancer incidence, respectively (*P* for modification < 0.05), while these relationships were persistently significant in the corresponding subgroups (Additional file 1: Table S3).

**Table 4:** Associations of liver function biomarkers with the risk of incident liver cancer (*N* = 356,894)

Liver function biomarker	HR (95% CI) for per 10-unit increase	HR (95% CI) according to liver function biomarkers concentration in quartiles				*P* trend
		Q1	Q2	Q3	Q4	
ALT (U/L)	**1.17 (1.15, 1.20)**	1 (Reference)	0.74 (0.45, 1.21)	1.26 (0.82, 1.94)	**3.25 (2.18, 4.84)**	** < 0.001**
AST (U/L)	**1.20 (1.18, 1.22)**	1 (Reference)	0.67 (0.40, 1.10)	0.99 (0.63, 1.55)	**3.55 (2.45, 5.15)**	** < 0.001**
TBIL (μmol//L)	**1.69 (1.47, 1.93)**	1 (Reference)	0.93 (0.64, 1.37)	1.03 (0.71, 1.50)	**1.68 (1.18, 2.38)**	**0.001**
GGT (U/L)	**1.06 (1.06, 1.07)**	1 (Reference)	0.95 (0.54, 1.67)	1.33 (0.79, 2.26)	**5.17 (3.22, 8.32)**	** < 0.001**
ALP (U/L)	**1.08 (1.07, 1.09)**	1 (Reference)	0.87 (0.58, 1.31)	1.14 (0.78, 1.67)	**2.12 (1.49, 3.00)**	** < 0.001**
TP (g/L)	**1.81 (1.37, 2.39)**	1 (Reference)	1.36 (0.94, 1.97)	1.35 (0.93, 1.96)	**2.13 (1.51, 3.01)**	** < 0.001**
ALB (g/L)	**0.29 (0.18, 0.46)**	1 (Reference)	0.79 (0.58, 1.07)	**0.54 (0.38, 0.77)**	**0.66 (0.47, 0.94)**	**0.002**

Q1, Q2, Q3, and Q4 refer to the 1st (the lowest; < 25th percentile), 2nd (25th ~ 50th percentile), 3rd (50th ~ 75th percentile), and 4th (the highest; ≥ 75th percentile) quartiles of liver function biomarkers

Models were adjusted for age, sex, ethnicity, BMI, smoking status, alcohol consumption, healthy diet, physical activity, any treatment/medication taken, Townsend deprivation index, family history of cancer, and CRP

*Abbreviations*: *HR* hazard ratio, *CI* confidence interval, *ALT* alanine transaminase, *AST* aspartate transaminase, *TBIL* total bilirubin, *GGT* gamma-glutamyl transferase, *ALP* alkaline phosphatase, *TP* total protein, *ALB* albumin

*P* trend was tested by including the quartile order of liver function biomarkers as a continuous variable in the model

### Sensitivity analysis

As shown in the supplementary materials (Additional file 1: Table S4 ~ Table S13), the associations of sleep or liver function biomarkers with liver cancer incidence did not change apparently after excluding participants with incident liver cancer in the first 2 years of follow-up, excluding individuals with liver disease at baseline, conducting additional model adjustment, performing IPW analysis, or conducting competing risk model.

### Joint associations of sleep and liver function biomarkers with liver cancer incidence

**Fig. 2:**
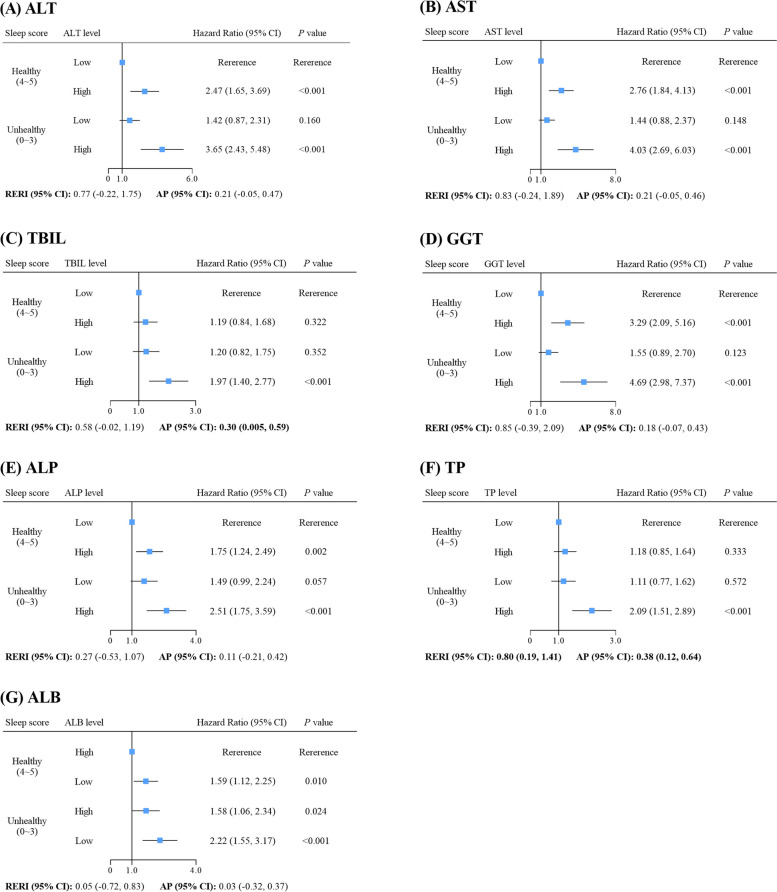
Risk of incident liver cancer according to sleep and liver function biomarkers (*N* = 356,894). Abbreviations: HR, hazard ratio; CI, confidence interval; RERI, relative excess risk due to the interaction; AP, attributable proportion due to the interaction; ALT, alanine transaminase; AST, aspartate transaminase; TBIL, total bilirubin; GGT, gamma-glutamyl transferase; ALP, alkaline phosphatase; TP, total protein; ALB, albumin. Median concentration was used as the cut-off concentration for high or low classification of the liver function biomarker. Models were adjusted for age, sex, ethnicity, BMI, smoking status, alcohol consumption, healthy diet, physical activity, any treatment/medication taken, Townsend deprivation index, family history of cancer, and CRP

Joint associations of unhealthy sleep and liver function biomarkers with liver cancer incidence were observed (Fig. 2). Individuals with unhealthy sleep and high level of ALT (HR = 3.65, 95% CI: 2.43, 5.48), AST (HR = 4.03, 95% CI: 2.69, 6.03), TBIL (HR = 1.97, 95% CI: 1.40, 2.77), GGT (HR = 4.69, 95% CI: 2.98, 7.37), ALP (HR = 2.51, 95% CI: 1.75, 3.59), or TP (HR = 2.09, 95% CI: 1.51, 2.89) or low level of ALB (HR = 2.22, 95% CI:1.55, 3.17) were at the highest risk for liver cancer incidence compared with those with healthy sleep and low level of ALT, AST, TBIL, GGT, ALP, or TP or high level of ALB. Positive additive interaction of unhealthy sleep with high TP level was indicated by the significant RERI and AP. For unhealthy sleep with a high TP level, the RERI was 0.80 (95% CI: 0.19, 1.41), which suggested that there would be a 0.80 relative excess risk from the additive interaction, accounting for 38% (AP = 0.38, 95% CI: 0.12, 0.64) of the risk of liver cancer incidence in individuals exposed to both unhealthy sleep and high TP level. Moreover, 30% (AP = 0.30, 95% CI: 0.005, 0.59) of the risk of liver cancer incidence was ascribed to the interaction of unhealthy sleep and high TBIL level.

## Discussion

In this study, we found that unhealthy sleep, particularly unfavorable sleep duration, insomnia, and snoring, was associated with an increased risk of liver cancer incidence. In addition, participants with higher levels of ALT, AST, TBIL, GGT, ALP, or TP or lower levels of ALB tend to have a higher risk of liver cancer incidence. The most substantial relative increase in the risk of liver cancer incidence was observed in individuals with unhealthy sleep and high levels of ALT, AST, TBIL, GGT, ALP, or TP or low levels of ALB. Furthermore, our study provided quantitative data regarding the additive interaction effect between unhealthy sleep and high TP levels on liver cancer incidence. Our findings contribute to the understanding of the relationships of sleep and liver function biomarkers with liver cancer incidence, and significant public health implications for the identification of susceptible populations and for the early prevention of liver cancer.

Accumulating evidence has demonstrated the adverse impact of unhealthy sleep on liver health [32–35]. Unhealthy sleep characteristics, including circadian rhythm abnormalities, sleep-disordered breathing, increased daytime somnolence, restless legs syndrome, nocturnal desaturations, and insomnia with nocturnal awakenings, could be frequently observed in chronic liver disease patients [32]. A crucial review summarized the epidemiology and pathophysiology of sleep–wake disturbance in liver disease, discussed the pathways involved, and indicated that altered melatonin metabolism, neuromuscular complications, and aberrant thermoregulation contributed to the development of liver disease driven by sleep–wake disorders [33]. However, evidence regarding the impact of sleep disturbance on liver cancer remains lacking. One of the few existing studies in this field was the Women’s Health Initiative Study conducted on 139,368 US postmenopausal women, and it found that sleep duration ≥ 9 h was associated with a moderately increased risk of liver cancer incidence in obese women [36]. In contrast, our study identified a significant association between unhealthy sleep, particularly unfavorable sleep duration, insomnia, and snoring, and increased risk of liver cancer incidence in a large prospective cohort with representative general population, systematic sleep evaluation, and relatively complete and precise diseases/death record. Notably, our study revealed a U-shaped relationship between sleep duration and liver cancer risk, indicating that both too short and too long sleep durations might be associated with increased risk of liver cancer incidence. At present, evidence on the impact of the sleep duration on the risk of cancer is still controversial. For instance, the U-shaped association between sleep duration and risk of colorectal or lung cancer was observed in large prospective cohort studies [8, 37]. On the other hand, there was no significant association of sleep duration with breast or prostate cancer risk according to published literature [38, 39]. The difference may be attributed to the different pathogeneses of these cancers, and disruption of the immune–inflammatory balance may be a reasonable biologic mechanism underlying the carcinogenic effect of short and long sleep durations [40, 41].

Moreover, our study revealed that ALT, AST, TBIL, GGT, ALP, and TP were positively associated with increased risk of liver cancer incidence while the association of ALB was inverse. Our findings were supported by most of the studies concerning the associations between liver function biomarkers and the risk of cancer or mortality [42–44]. According to published prospective cohort studies assessing the associations of these liver function biomarkers with all-cause mortality in the general populations, GGT and ALP were associated with increased all-cause mortality, while there were geographical variations in the association of ALT with all-cause mortality [45]. In addition, elevated GGT and AST/ALT were independent risk factors for predicting overall survival rate in primary hepatic carcinoma patients, and elevated levels of these biomarkers suggested poor prognosis [18]. Moreover, the inverse association of ALB with liver cancer incidence observed in our study was also generally supported by previous findings on the inverse associations of ALB with liver disease and liver cancer [46–48], as well as with other cancers [49–51]. As a cohort study of 82,061 Chinese participants found, ALB level was inversely associated with the incident risk of overall, lung, colorectal, and liver cancer in linear dose-dependent manners [46]. What's more, several studies indicated the therapeutic implications of human serum ALB due to its wide range of important physiologic functions, including oncotic effect, immunomodulation, antioxidant effect, endothelial stabilization, and binding to multiple drugs, toxins, and other molecules [47, 48]. Our findings of the significant relationships between these routine liver function biomarkers and risk of liver cancer incidence indicated the potential protective effects of controlling the levels of these liver function biomarkers, and it may be helpful in screening people at high liver cancer risk during routine liver function tests.

Furthermore, we conducted a novel investigation into the joint associations of sleep and liver function biomarkers with liver cancer incidence, as well as potential additive interactions. Our findings showed that individuals with unhealthy sleep and high levels of ALT, AST, TBIL, GGT, ALP, or TP or low level of ALB had a significantly increased risk of liver cancer incidence compared to those with healthy sleep and low levels of ALT, AST, TBIL, GGT, ALP, or TP or high level of ALB. Moreover, the joint effect of unhealthy sleep and high level of TP was greater than the sum of the two individual effects, and 38% of liver cancer risk could be attributed to the additive interaction. Our findings could aid in highlighting the joint associations of sleep and liver function biomarkers with liver cancer risk, and significant public health implications for the identification of susceptible populations and for the early prevention of liver cancer. Specifically, adhering to a healthy sleep and controlling routine liver function biomarkers levels will be beneficial in reducing liver cancer incidence risk.

Our study has several significant strengths, including the large nationwide general population sample, prospective study design, systematic assessment of sleep, uniform measurement of biomarkers, adequate adjustment for potential confounders, and relatively complete diseases/death records. In addition, we demonstrated the compelling associations of sleep and liver function biomarkers and their joint association with the risk of liver cancer incidence, providing potent evidence that adhering to healthy sleep and controlling liver function biomarkers levels may be a promising strategy for reducing liver cancer incidence risk. Moreover, we comprehensively investigated the association between sleep and liver cancer in terms of overall sleep score and independent sleep traits and found that unhealthy sleep, particularly unfavorable sleep duration, insomnia, and snoring, increased the risk of liver cancer incidence. However, our study also has some limitations. First, only the liver function biomarkers levels at baseline were used in the current study, which may be subject to random error and may lead to the misclassification of these biomarkers across the long follow-up period. Nevertheless, the reproductivities of these liver function biomarkers have been identified by previous studies to be fair to excellent so that the liver function biomarkers at baseline can also be representative and acceptable and capable of capturing the long-term effect on liver cancer [16]. Second, the incidence of liver cancer in this study is limited. Therefore, although the results of this study are robust, more studies conducted in populations with higher incidence of liver cancer are encouraged to validate our findings. Finally, as an observational study, we could not exclude the potential inverse association and residual confoundings, even though we scientifically adjusted for potential confounders as much as possible and conducted essential sensitivity analyses.

## Conclusions

Unhealthy sleep, as well as increased levels of ALT, AST, TBIL, GGT, ALP, and TP and decreased levels of ALB, were significantly associated with increased risk of liver cancer incidence. Participants with unhealthy sleep and jointly had lower levels of ALB or higher levels of ALT, AST, TBIL, GGT, ALP, or particularly TP were at higher risk of liver cancer incidence. Our findings suggest that adhering to healthy sleep and controlling liver function biomarker levels may be a promising strategy for reducing the risk of liver cancer incidence.

### Supplementary Information

Additional file 1: Table S1. Definition of the sleep traits and healthy sleep scoring system in the UK Biobank. Table S2. Association of sleep score with the risk of incident liver cancer (*N* = 356,850). Table S3. Stratified analyses of the associations between liver function biomarkers and the risk of incident liver cancer (*N* = 356,894). Table S4. Association of sleep with the risk of incident liver cancer after excluding incident liver cancer in the first 2 years of follow-up (*N* = 356,850). Table S5. Associations of liver function biomarkers with the risk of incident liver cancer after excluding incident liver cancer in the first 2 years of follow-up (*N* = 356,850). Table S6. Association of sleep with the risk of incident liver cancer after excluding individuals with liver disease at baseline (*N* = 356,858). Table S7. Associations of liver function biomarkers with the risk for incident liver cancer after excluding individuals with liver disease at baseline (*N* = 356,858). Table S8. Association of sleep with the risk of incident liver cancer after further adjustment (N = 356,894). Table S9. Associations of liver function biomarkers with the risk for incident liver cancer after further adjustment (*N* = 356,894). Table S10. Association of sleep with the risk of incident liver cancer by inverse probability weighting analysis (N = 356,894). Table S11. Associations of liver function biomarkers with the risk for incident liver cancer by inverse probability weighting analysis (*N* = 356,894). Table S12. Association of sleep with the risk of incident liver cancer in mortality competing risk model (*N* = 356,894). Table S13. Associations of liver function biomarkers with the risk for incident liver cancer in mortality competing risk model (*N* = 356,894). Fig. S1. Kaplan–Meier curves of sleep and independent sleep traits with incident liver cancer. Fig. S2. Association of sleep duration with the risk of incident liver cancer across different hours (*N* = 356,894). Fig. S3. Associations between liver function biomarkers and the risk of incident liver cancer by restricted cubic spline regression (*N* = 356,894)

## Data Availability

The datasets generated and/or analyzed during the current study are available from the corresponding author on reasonable request.

## Abbreviations

ALB: Albumin
ALP: Alkaline phosphatase
ALT: Alanine transaminase
AP: Attributable proportion due to the interaction
AST: Aspartate transaminase
BMI: Body mass index
CI: Confidence interval
CRP: C-reaction protein
GGT: Gamma-glutamyl transferase
HCC: Hepatocellular carcinoma
HR: Hazard ratio
ICD: International classification of diseases
RERI: Relative excess risk due to the interaction
SD: Standard deviation
TBIL: Total bilirubin
TP: Total protein

## References

[1] Sung H, Ferlay J, Siegel RL, Laversanne M, Soerjomataram I, Jemal A, Bray F (2021). Global cancer statistics 2020: GLOBOCAN estimates of incidence and mortality worldwide for 36 cancers in 185 countries. CA Cancer J Clin.

[2] Rumgay H, Arnold M, Ferlay J, Lesi O, Cabasag CJ, Vignat J, Laversanne M, McGlynn KA, Soerjomataram I (2022). Global burden of primary liver cancer in 2020 and predictions to 2040. J Hepatol.

[3] Grewal P, Viswanathen VA (2012). Liver cancer and alcohol. Clin Liver Dis.

[4] Yang W-S, Zeng X-F, Liu Z-N, Zhao Q-H, Tan Y-T, Gao J, Li H-L, Xiang Y-B (2020). Diet and liver cancer risk: a narrative review of epidemiological evidence. Br J Nutr.

[5] Marengo A, Rosso C, Bugianesi E (2016). Liver cancer: connections with obesity, fatty liver, and cirrhosis. Annu Rev Med.

[6] Zelber-Sagi S, Noureddin M, Shibolet O (2021). Lifestyle and hepatocellular carcinoma what is the evidence and prevention recommendations. Cancers (Basel).

[7] Chaput J-P, Gray CE, Poitras VJ, Carson V, Gruber R, Olds T, Weiss SK, Connor Gorber S, Kho ME, Sampson M (2016). Systematic review of the relationships between sleep duration and health indicators in school-aged children and youth. Appl Physiol Nutr Metab.

[8] Xie J, Zhu M, Ji M, Fan J, Huang Y, Wei X, Jiang X, Xu J, Yin R, Wang Y (2021). Relationships between sleep traits and lung cancer risk: a prospective cohort study in UK Biobank. Sleep.

[9] Zhang X, Giovannucci EL, Wu K, Gao X, Hu F, Ogino S, Schernhammer ES, Fuchs CS, Redline S, Willett WC (2013). Associations of self-reported sleep duration and snoring with colorectal cancer risk in men and women. Sleep.

[10] Song C, Zhang R, Wang C, Fu R, Song W, Dou K, Wang S (2021). Sleep quality and risk of cancer: findings from the English longitudinal study of aging. Sleep.

[11] Shi T, Min M, Sun C, Zhang Y, Liang M, Sun Y (2020). Does insomnia predict a high risk of cancer? A systematic review and meta-analysis of cohort studies. J Sleep Res.

[12] Mogavero MP, DelRosso LM, Fanfulla F, Bruni O, Ferri R (2021). Sleep disorders and cancer: state of the art and future perspectives. Sleep Med Rev.

[13] Ning D, Fang Y, Zhang W. Association of habitual sleep duration and its trajectory with the risk of cancer according to sex and body mass index in a population-based cohort. Cancer. 2023;129(22):3582–94. 10.1002/cncr.34951 37432142

[14] Liu W, Cao S, Shi D, Yu L, Qiu W, Chen W, Wang B (2023). Single-chemical and mixture effects of multiple volatile organic compounds exposure on liver injury and risk of non-alcoholic fatty liver disease in a representative general adult population. Chemosphere.

[15] Newsome PN, Cramb R, Davison SM, Dillon JF, Foulerton M, Godfrey EM, Hall R, Harrower U, Hudson M, Langford A (2018). Guidelines on the management of abnormal liver blood tests. Gut.

[16] He MM, Fang Z, Hang D, Wang F, Polychronidis G, Wang L, Lo CH, Wang K, Zhong R, Knudsen MD (2021). Circulating liver function markers and colorectal cancer risk: a prospective cohort study in the UK Biobank. Int J Cancer.

[17] Li J, Tao H, Zhang E, Huang Z (2021). Diagnostic value of gamma-glutamyl transpeptidase to alkaline phosphatase ratio combined with gamma-glutamyl transpeptidase to aspartate aminotransferase ratio and alanine aminotransferase to aspartate aminotransferase ratio in alpha-fetoprotein-negative hepatocellular carcinoma. Cancer Med.

[18] Zhang L-X, Lv Y, Xu AM, Wang H-Z (2019). The prognostic significance of serum gamma-glutamyltransferase levels and AST/ALT in primary hepatic carcinoma. BMC Cancer.

[19] Sudlow C, Gallacher J, Allen N, Beral V, Burton P, Danesh J, Downey P, Elliott P, Green J, Landray M (2015). UK Biobank: an open access resource for identifying the causes of a wide range of complex diseases of middle and old age. PLoS Med.

[20] Fan M, Sun D, Zhou T, Heianza Y, Lv J, Li L, Qi L (2020). Sleep patterns, genetic susceptibility, and incident cardiovascular disease: a prospective study of 385 292 UK biobank participants. Eur Heart J.

[21] Li X, Xue QC, Wang MY, Zhou T, Ma H, Heianza Y, Qi L (2021). Adherence to a healthy sleep pattern and incident heart failure a prospective study of 408 802 UK Biobank participants. Circulation.

[22] Huang B-H, Duncan MJ, Cistulli PA, Nassar N, Hamer M, Stamatakis E (2022). Sleep and physical activity in relation to all-cause, cardiovascular disease and cancer mortality risk. Br J Sports Med.

[23] UK Biobank Biomarker Project - Companion document to accompany serum biomarker data. Prepared for: UK Biobank Showcase. Version 1.0. 2019. https://biobank.ndph.ox.ac.uk/showcase/showcase/docs/serum_biochemistry.pdf.

[24] Ford JC, O'Rourke K, Veinot JP, Walley VM (2000). Histologic estimation of coronary artery stenoses: reproducibility and the effect of training. Cardiovasc Pathol.

[25] Du W, Guan H, Wan X, Zhu Z, Yu H, Luo P, Chen L, Su J, Lu Y, Hang D (2023). Circulating liver function markers and the risk of COPD in the UK Biobank. Front Endocrinol (Lausanne).

[26] Chen J, Dan L, Tu X, Sun Y, Deng M, Chen X, Hesketh T, Li R, Wang X, Li X (2023). Metabolic dysfunction-associated fatty liver disease and liver function markers are associated with Crohn's disease but not Ulcerative Colitis: a prospective cohort study. Hepatol Int.

[27] Murphy N, Carreras-Torres R, Song MY, Chan AT, Martin RM, Papadimitriou N, Dimou N, Tsilidis KK, Banbury B, Bradbury KE (2020). Circulating levels of insulin-like growth factor 1 and insulin-like growth factor binding protein 3 associate with risk of colorectal cancer based on serologic and mendelian randomization analyses. Gastroenterology.

[28] Greenland S (1989). Modeling and variable selection in epidemiologic analysis. Am J Public Health.

[29] Said MA, Verweij N, van der Harst P (2018). Associations of combined genetic and lifestyle risks with incident cardiovascular disease and diabetes in the UK Biobank Study. JAMA Cardiol.

[30] Guidelines for data processing and analysis of the International Physical Activity Questionnaire (IPAQ) – short and long forms. 2005. https://biobank.ndph.ox.ac.uk/showcase/ukb/docs/ipaq_analysis.pdf.

[31] Assmann SF, Hosmer DW, Lemeshow S, Mundt KA (1996). Confidence intervals for measures of interaction. Epidemiology.

[32] De Cruz S, Espiritu JRD, Zeidler M, Wang TS (2012). Sleep disorders in chronic liver disease. Semin Respir Crit Care Med.

[33] Marjot T, Ray DW, Williams FR, Tomlinson JW, Armstrong MJ (2021). Sleep and liver disease: a bidirectional relationship. Lancet Gastroenterol Hepatol.

[34] Plotogea O-M, Ilie M, Bungau S, Chiotoroiu AL, Stanescu AMA, Diaconu CC (2021). Comprehensive overview of sleep disorders in patients with chronic liver disease. Brain Sci.

[35] Shen N, Wang P, Yan W (2016). Sleep duration and the risk of fatty liver disease: a systematic review and meta-analysis. Sci Rep.

[36] Royse KE, El-Serag HB, Chen L, White DL, Hale L, Sangi-Haghpeykar H, Jiao L (2017). Sleep duration and risk of liver cancer in postmenopausal women: the women's health initiative study. J Womens Health (Larchmt).

[37] Jiao L, Duan Z, Sangi-Haghpeykar H, Hale L, White DL, El-Serag HB (2013). Sleep duration and incidence of colorectal cancer in postmenopausal women. Br J Cancer.

[38] Markt SC, Flynn-Evans EE, Valdimarsdottir UA, Sigurdardottir LG, Tamimi RM, Batista JL, Haneuse S, Lockley SW, Stampfer M, Wilson KM (2016). Sleep duration and disruption and prostate cancer risk: a 23-year prospective study. Cancer Epidemiol Biomarkers Prev.

[39] Qin Y, Zhou Y, Zhang X, Wei X, He J (2014). Sleep duration and breast cancer risk: a meta-analysis of observational studies. Int J Cancer.

[40] Irwin MR, Olmstead R, Carroll JE (2016). Sleep disturbance, sleep duration, and inflammation: a systematic review and meta-analysis of cohort studies and experimental sleep deprivation. Biol Psychiatry.

[41] Bollinger T, Bollinger A, Oster H, Solbach W (2010). Sleep, immunity, and circadian clocks: a mechanistic model. Gerontology.

[42] Koehler EM, Sanna D, Hansen BE, van Rooij FJ, Heeringa J, Hofman A, Tiemeier H, Stricker BH, Schouten JNL, Janssen HLA (2014). Serum liver enzymes are associated with all-cause mortality in an elderly population. Liver Int.

[43] Strasak AM, Rapp K, Brant LJ, Hilbe W, Gregory M, Oberaigner W, Ruttmann E, Concin H, Diem G, Pfeiffer KP (2008). Association of gamma-glutamyltransferase and risk of cancer incidence in men: a prospective study. Cancer Res.

[44] Katzke V, Johnson T, Sookthai D, Hüsing A, Kühn T, Kaaks R (2020). Circulating liver enzymes and risks of chronic diseases and mortality in the prospective EPIC-Heidelberg case-cohort study. BMJ Open.

[45] Kunutsor SK, Apekey TA, Seddoh D, Walley J (2014). Liver enzymes and risk of all-cause mortality in general populations: a systematic review and meta-analysis. Int J Epidemiol.

[46] Yang Z, Zheng Y, Wu Z, Wen Y, Wang G, Chen S, Tan F, Li J, Wu S, Dai M (2021). Association between pre-diagnostic serum albumin and cancer risk: results from a prospective population-based study. Cancer Med.

[47] Spinella R, Sawhney R, Jalan R (2016). Albumin in chronic liver disease: structure, functions and therapeutic implications. Hepatol Int.

[48] Jagdish RK, Maras JS, Sarin SK (2021). Albumin in advanced liver diseases: the good and bad of a drug!. Hepatology.

[49] Stevens RG, Jones DY, Micozzi MS, Taylor PR (1988). Body iron stores and the risk of cancer. N Engl J Med.

[50] Kühn T, Sookthai D, Graf ME, Schübel R, Freisling H, Johnson T, Katzke V, Kaaks R (2017). Albumin, bilirubin, uric acid and cancer risk: results from a prospective population-based study. Br J Cancer.

[51] Ko WF, Helzlsouer KJ, Comstock GW (1994). Serum albumin, bilirubin, and uric acid and the anatomic site-specific incidence of colon cancer. J Natl Cancer Inst.

