# Supplementary material for: Sleep and liver function biomarkers in relation to risk of incident liver cancer: a nationwide prospective cohort study
Source: BMC Med. 2024 Jun 24;22:261. doi: 10.1186/s12916-024-03440-w (PMC11197319; doi:10.1186/s12916-024-03440-w)
Supplement: Supplementary file 1 — Additional file 1: Table S1. Definition of the sleep traits and healthy sleep scoring system in the UK Biobank. Table S2. Association of sleep score with the risk of incident liver cancer (N = 356,850). Table S3. Stratified analyses of the associations between liver function biomarkers and the risk of incident liver cancer (N = 356,894). Table S4. Association of sleep with the risk of incident liver cancer after excluding incident liver cancer in the first 2 years of follow-up (N = 356,850). Table S5. Associations of liver function biomarkers with the risk of incident liver cancer after excluding incident liver cancer in the first 2 years of follow-up (N = 356,850). Table S6. Association of sleep with the risk of incident liver cancer after excluding individuals with liver disease at baseline (N = 356,858). Table S7. Associations of liver function biomarkers with the risk for incident liver cancer after excluding individuals with liver disease at baseline (N = 356,858). Table S8. Association of sleep with the risk of incident liver cancer after further adjustment (N = 356,894). Table S9. Associations of liver function biomarkers with the risk for incident liver cancer after further adjustment (N = 356,894). Table S10. Association of sleep with the risk of incident liver cancer by inverse probability weighting analysis (N = 356,894). Table S11. Associations of liver function biomarkers with the risk for incident liver cancer by inverse probability weighting analysis (N = 356,894). Table S12. Association of sleep with the risk of incident liver cancer in mortality competing risk model (N = 356,894). Table S13. Associations of liver function biomarkers with the risk for incident liver cancer in mortality competing risk model (N = 356,894). Fig. S1. Kaplan–Meier curves of sleep and independent sleep traits with incident liver cancer. Fig. S2. Association of sleep duration with the risk of incident liver cancer across different hours (N = 356,894). Fig. S3. Associations [file 12916_2024_3440_MOESM1_ESM.docx]

**Sleep and liver function biomarkers in relation to risk of incident liver cancer: A nationwide prospective cohort study**

**Additional file 1**

**Table/Figure legends**

**Table S1.** Definition of the sleep traits and healthy sleep scoring system in the UK Biobank.

**Table S2.** Association of sleep score with the risk of incident liver cancer (N=356,850)

**Table S3.** Stratified analyses of the associations between liver function biomarkers and the risk of incident liver cancer (N=356,894)

**Table S4.** Association of sleep with the risk of incident liver cancer after excluding incident liver cancer in the first 2 years of follow-up (N=356,850)

**Table S5.** Associations of liver function biomarkers with the risk of incident liver cancer after excluding incident liver cancer in the first 2 years of follow-up (N=356,850)

**Table S6.** Association of sleep with the risk of incident liver cancer after excluding individuals with liver disease at baseline (N=356,858)

**Table S7.** Associations of liver function biomarkers with the risk for incident liver cancer after excluding individuals with liver disease at baseline (N=356,858)

**Table S8.** Association of sleep with the risk of incident liver cancer after further adjustment (N=356894)

**Table S9.** Associations of liver function biomarkers with the risk for incident liver cancer after further adjustment (N=356894).

**Table S10.** Association of sleep with the risk of incident liver cancer by inverse probability weighting analysis (N=356,894)

**Table S11.** Associations of liver function biomarkers with the risk for incident liver cancer by inverse probability weighting analysis (N=356,894)

**Table S12.** Association of sleep with the risk of incident liver cancer in mortality competing risk model (N=356,894)

**Table S13**. Associations of liver function biomarkers with the risk for incident liver cancer in mortality competing risk model (N=356,894)

**Fig. S1.** Kaplan-Meier curves of sleep and independent sleep traits with incident liver cancer (N=356,894).

**Fig. S2.** Association of sleep duration with the risk of incident liver cancer across different hours (N=356,894).

**Fig. S3.** Associations between liver function biomarkers and the risk of incident liver cancer by restricted cubic spline regression (N=356,894).

**Supplementary Materials**

**Table S1.** Definition of the sleep traits and healthy sleep scoring system in the UK Biobank.

| Sleep traits | UK Biobank Code | UK Biobank questionnaire | Healthy answer | Unhealthy answer |
| --- | --- | --- | --- | --- |
| Sleep Duration | 1160 | About how many hours sleep do you get in every 24 hours? (please include naps) | 7-8 hours/day | <7 or >8 hours/day |
| Morning/evening chronotype | 1180 | Do you consider yourself to be? | Definitely a "morning" person; More a "morning" than "evening" person. | Definitely an "evening" person; More an "evening" than a "morning person. |
| Insomnia | 1200 | Do you have trouble falling asleep at night or do you wake up in the middle of the night? | Never/rarely; Sometimes | Usually |
| Snoring | 1210 | Does your partner or a close relative or friend complain about your snoring? | No | Yes |
| Excessive daytime sleepiness | 1220 | How likely are you to doze off or fall asleep during the daytime when you don't mean to? (e.g. when working, reading or driving) | Never/rarely; Sometimes | Often; All the Time |

**Table S2**. Association of sleep score with the risk of incident liver cancer (N=356,850)

| Model | HR (95% CI) of incident liver cancer for per 1-unit increase in sleep score | *P* |
| --- | --- | --- |
| Model 1 | 1.17 (1.07, 1.29) | 0.001 |
| Model 2 | 1.23 (1.10, 1.36) | <0.001 |
| Model 3 | 1.24 (1.11, 1.39) | <0.001 |

Abbreviations: HR, hazard ratio; CI, confidence interval.

Model 1 was a crude model; Model 2 was adjusted for age, sex, ethnic, BMI, smoking status, alcohol consumption, healthy diet, and physical activity; Model 3 was adjusted for age, sex, ethnic, BMI, smoking status, alcohol consumption, healthy diet, physical activity, any treatment/medication taken, Townsend deprivation index, family history of cancer, and CRP.

**Table S3.** Stratified analyses of the associations between liver function biomarkers and the risk of incident liver cancer (N=356,894)

| Characteristic | HR (95% CI) for per 10-unit increase in liver function biomarkers | | | | | | |
| --- | --- | --- | --- | --- | --- | --- | --- |
|  | ALT (U/L) | AST (U/L) | TBIL (μmol//L) | GGT (U/L) | ALP (U/L) | TP (g/L) | ALB (g/L) |
| Age |  |  |  |  |  |  |  |
| <60 years | 1.18 (1.15, 1.21) | 1.21 (1.18, 1.24) | 1.86 (1.57, 2.22) | 1.07 (1.06, 1.07) | 1.09 (1.08, 1.11) | 1.71 (1.08, 2.71) | 0.19 (0.10, 0.38) |
| ≥60 years | 1.18 (1.15, 1.22) | 1.21 (1.18, 1.25) | 1.54 (1.22, 1.94) | 1.06 (1.05, 1.07) | 1.07 (1.04, 1.09) | 1.90 (1.34, 2.69) | 0.37 (0.20, 0.67) |
| *P* for modification | 0.592 | 0.464 | 0.231 | 0.368 | 0.084 | 0.572 | 0.296 |
| Sex |  |  |  |  |  |  |  |
| Female | 1.13 (1.08, 1.18) | 1.17 (1.13, 1.22) | 1.82 (1.17, 2.81) | 1.06 (1.04, 1.07) | 1.07 (1.05, 1.09) | 1.28 (0.75, 2.18) | 0.67 (0.27, 1.62) |
| Male | 1.19 (1.16, 1.21) | 1.21 (1.19, 1.23) | 1.69 (1.45, 1.96) | 1.06 (1.06, 1.07) | 1.09 (1.08, 1.11) | 2.06 (1.48, 2.85) | 0.21 (0.13, 0.35) |
| *P* for modification | 0.105 | 0.246 | 0.604 | 0.197 | 0.146 | 0.109 | **0.018** |
| BMI |  |  |  |  |  |  |  |
| <25kg/m^2^ | 1.20 (1.16, 1.25) | 1.16 (1.12, 1.20) | 1.10 (0.66, 1.84) | 1.06 (1.05, 1.08) | 1.09 (1.07, 1.11) | 2.14 (1.25, 3.67) | 0.32 (0.13, 0.82) |
| ≥25kg/m^2^ | 1.17 (1.15, 1.20) | 1.24 (1.22, 1.27) | 1.77 (1.56, 2.01) | 1.06 (1.06, 1.07) | 1.08 (1.07, 1.10) | 1.73 (1.25, 2.39) | 0.29 (0.17, 0.48) |
| *P* for modification | 0.151 | **0.002** | 0.449 | 0.759 | 0.536 | 0.354 | 0.578 |
| Smoking status |  |  |  |  |  |  |  |
| Never | 1.16 (1.12, 1.20) | 1.19 (1.15, 1.22) | 1.78 (1.42, 2.23) | 1.07 (1.06, 1.08) | 1.09 (1.08, 1.11) | 1.45 (0.89, 2.36) | 0.32 (0.14, 0.72) |
| Former | 1.19 (1.16, 1.21) | 1.24 (1.21, 1.27) | 1.72 (1.44, 2.05) | 1.06 (1.05, 1.07) | 1.06 (1.04, 1.08) | 1.93 (1.30, 2.85) | 0.35 (0.18, 0.67) |
| Current | 1.28 (1.22, 1.36) | 1.18 (1.13, 1.23) | 2.91 (2.10, 4.04) | 1.06 (1.05, 1.07) | 1.14 (1.09, 1.20) | 2.81 (1.48, 5.34) | 0.29 (0.09, 0.92) |
| *P* for modification | **0.015** | 0.784 | 0.151 | 0.227 | 0.172 | 0.066 | 0.917 |
| Alcohol consumption |  |  |  |  |  |  |  |
| Never/Seldom | 1.17 (1.14, 1.20) | 1.23 (1.20, 1.27) | 1.74 (1.45, 2.09) | 1.07 (1.06, 1.08) | 1.09 (1.07, 1.12) | 1.49 (0.93, 2.38) | 0.22 (0.10, 0.46) |
| 1-4 times per week | 1.17 (1.14, 1.21) | 1.19 (1.16, 1.22) | 1.73 (1.35, 2.23) | 1.07 (1.06, 1.08) | 1.07 (1.05, 1.09) | 1.90 (1.23, 2.93) | 0.40 (0.19, 0.84) |
| Almost daily | 1.20 (1.16, 1.25) | 1.20 (1.16, 1.24) | 2.13 (1.57, 2.88) | 1.06 (1.05, 1.06) | 1.07 (1.05, 1.10) | 1.99 (1.14, 3.47) | 0.42 (0.16, 1.08) |
| *P* for modification | 0.285 | 0.167 | 0.346 | **0.037** | 0.366 | 0.366 | 0.303 |

Abbreviations: HR, hazard ratio; CI, confidence interval; ALT, alanine transaminase; AST, aspartate transaminase; TBIL, total bilirubin; GGT, gamma-glutamyl transferase; ALP, alkaline phosphatase; TP, total protein; ALB, albumin.

Models were adjusted for age, sex, ethnic, BMI, smoking status, alcohol consumption, healthy diet, physical activity, any treatment/medication taken, Townsend deprivation index, family history of cancer, and CRP.

*P* for interaction was calculated by adding a product term of the level of liver function biomarker and the stratified variable into the statistic model.

**Table S4.** Association of sleep with the risk of incident liver cancer after excluding incident liver cancer in the first 2 years of follow-up (N=356,850)

| Sleep | Model 1 | Model 2 | Model 3 |
| --- | --- | --- | --- |
|  | HR (95% CI) | HR (95% CI) | HR (95% CI) |
| Unhealthy sleep (sleep score: 0~3) | **1.42 (1.15, 1.75)** | **1.52 (1.19, 1.93)** | **1.58 (1.22, 2.04)** |
| Individual component |  |  |  |
| Unfavorable sleep duration | **1.50 (1.21, 1.85)** | **1.32 (1.03, 1.69)** | **1.34 (1.03, 1.74)** |
| Evening chronotype | 1.09 (0.84, 1.43) | 1.36 (0.99, 1.87) | 1.25 (0.89, 1.77) |
| Insomnia | **1.53 (1.23, 1.90)** | **1.39 (1.08, 1.78)** | **1.47 (1.13, 1.92)** |
| Snoring | 0.88 (0.71, 1.09) | **1.33 (1.03, 1.71)** | **1.41 (1.07, 1.84)** |
| Excessive daytime sleepiness | 1.66 (0.99, 2.79) | 1.25 (0.70, 2.24) | 1.37 (0.76, 2.46) |

Abbreviations: HR, hazard ratio; CI, confidence interval.

Model 1 was a crude model; Model 2 was adjusted for age, sex, ethnic, BMI, smoking status, alcohol consumption, healthy diet, and physical activity; Model 3 was adjusted for age, sex, ethnic, BMI, smoking status, alcohol consumption, healthy diet, physical activity, any treatment/medication taken, Townsend deprivation index, family history of cancer, and CRP.

**Table S5.** Associations of liver function biomarkers with the risk of incident liver cancer after excluding incident liver cancer in the first 2 years of follow-up (N=356,850)

| Liver function biomarkers | HR (95% CI) for per 10-unit increase | HR (95% CI) according to liver function biomarkers concentrations in quartiles | | | | *P* trend |
| --- | --- | --- | --- | --- | --- | --- |
|  |  | Q1 | Q2 | Q3 | Q4 |  |
| ALT (U/L) | **1.18 (1.16, 1.20)** | 1 (reference) | 0.61 (0.36, 1.03) | 0.97 (0.61, 1.53) | **2.93 (1.94, 4.41)** | **<0.001** |
| AST (U/L) | **1.20 (1.18, 1.22)** | 1 (reference) | 0.69 (0.41, 1.17) | 0.84 (0.52, 1.38) | **3.45 (2.33, 5.12)** | **<0.001** |
| TBIL (μmol//L) | **1.78 (1.52, 2.09)** | 1 (reference) | 0.89 (0.58, 1.35) | 1.10 (0.73, 1.64) | **1.69 (1.16, 2.46)** | **0.001** |
| GGT (U/L) | **1.06 (1.06, 1.07)** | 1 (reference) | 0.94 (0.52, 1.69) | 1.20 (0.69, 2.10) | **4.55 (2.75, 7.50)** | **<0.001** |
| ALP (U/L) | **1.08 (1.07, 1.09)** | 1 (reference) | 0.75 (0.48, 1.16) | 1.14 (0.76, 1.70) | **1.95 (1.34, 2.82)** | **<0.001** |
| TP (g/L) | **1.80 (1.33, 2.43)** | 1 (reference) | 1.33 (0.90, 1.97) | 1.27 (0.85, 1.89) | **2.05 (1.42, 2.95)** | **<0.001** |
| ALB (g/L) | **0.28 (0.17, 0.46)** | 1 (reference) | 0.77 (0.56, 1.07) | **0.60 (0.42, 0.86)** | **0.61 (0.42, 0.89)** | **0.003** |

Abbreviations: HR, hazard ratio; CI, confidence interval; ALT, alanine transaminase; AST, aspartate transaminase; TBIL, total bilirubin; GGT, gamma-glutamyl transferase; ALP, alkaline phosphatase; TP, total protein; ALB, albumin.

Models were adjusted for age, sex, ethnic, BMI, smoking status, alcohol consumption, healthy diet, physical activity, any treatment/medication taken, Townsend deprivation index, family history of cancer, and CRP.

*P* trend was tested by including the quartile order of liver function biomarkers as a continuous variable in the model.

**Table S6.** Association of sleep with the risk of incident liver cancer after excluding individuals with liver disease at baseline (N=356,858)

| Sleep | Model 1 | Model 2 | Model 3 |
| --- | --- | --- | --- |
|  | HR (95% CI) | HR (95% CI) | HR (95% CI) |
| Unhealthy sleep (sleep score: 0~3) | **1.35 (1.11, 1.64)** | **1.43 (1.14, 1.79)** | **1.46 (1.15, 1.85)** |
| Individual component |  |  |  |
| Unfavorable sleep duration | **1.48 (1.21, 1.81)** | **1.30 (1.03, 1.63)** | **1.29 (1.01, 1.65)** |
| Evening chronotype | 1.07 (0.83, 1.38) | 1.29 (0.95, 1.74) | 1.18 (0.85, 1.64) |
| Insomnia | **1.51 (1.23, 1.86)** | **1.37 (1.09, 1.74)** | **1.43 (1.11, 1.83)** |
| Snoring | 0.85 (0.70, 1.04) | 1.23 (0.98, 1.55) | **1.30 (1.01, 1.67)** |
| Excessive daytime sleepiness | 1.57 (0.95, 2.59) | 1.18 (0.68, 2.07) | 1.28 (0.73, 2.25) |

Abbreviations: HR, hazard ratio; CI, confidence interval.

Model 1 was a crude model; Model 2 was adjusted for age, sex, ethnic, BMI, smoking status, alcohol consumption, healthy diet, and physical activity; Model 3 was adjusted for age, sex, ethnic, BMI, smoking status, alcohol consumption, healthy diet, physical activity, any treatment/medication taken, Townsend deprivation index, family history of cancer, and CRP.

**Table S7.** Associations of liver function biomarkers with the risk for incident liver cancer after excluding individuals with liver disease at baseline (N=356,858)

| Liver function biomarkers | HR (95% CI) for per 10-unit increase | HR (95% CI) according to liver function biomarkers concentrations in quartiles | | | | *P* trend |
| --- | --- | --- | --- | --- | --- | --- |
|  |  | Q1 | Q2 | Q3 | Q4 |  |
| ALT (U/L) | **1.17 (1.15, 1.20)** | 1 (reference) | 0.74 (0.45, 1.21) | 1.26 (0.82, 1.94) | **3.25 (2.18, 4.84)** | **<0.001** |
| AST (U/L) | **1.20 (1.18, 1.22)** | 1 (reference) | 0.67 (0.40, 1.10) | 0.99 (0.63, 1.55) | **3.55 (2.45, 5.15)** | **<0.001** |
| TBIL (μmol//L) | **1.69 (1.47, 1.93)** | 1 (reference) | 0.93 (0.64, 1.37) | 1.03 (0.71, 1.50) | **1.68 (1.18, 2.38)** | **0.001** |
| GGT (U/L) | **1.06 (1.06, 1.07)** | 1 (reference) | 0.95 (0.54, 1.67) | 1.33 (0.79, 2.26) | **5.17 (3.22, 8.32)** | **<0.001** |
| ALP (U/L) | **1.08 (1.07, 1.09)** | 1 (reference) | 0.87 (0.58, 1.31) | 1.14 (0.78, 1.67) | **2.12 (1.49, 3.00)** | **<0.001** |
| TP (g/L) | **1.81 (1.37, 2.39)** | 1 (reference) | 1.36 (0.94, 1.97) | 1.35 (0.93, 1.96) | **2.13 (1.51, 3.01)** | **<0.001** |
| ALB (g/L) | **0.29 (0.18, 0.46)** | 1 (reference) | 0.79 (0.58, 1.07) | **0.54 (0.38, 0.77)** | **0.66 (0.47, 0.94)** | **0.003** |

Abbreviations: HR, hazard ratio; CI, confidence interval; ALT, alanine transaminase; AST, aspartate transaminase; TBIL, total bilirubin; GGT, gamma-glutamyl transferase; ALP, alkaline phosphatase; TP, total protein; ALB, albumin.

Models were adjusted for age, sex, ethnic, BMI, smoking status, alcohol consumption, healthy diet, physical activity, any treatment/medication taken, Townsend deprivation index, family history of cancer, and CRP.

*P* trend was tested by including the quartile order of liver function biomarkers as a continuous variable in the model.

**Table S8.** Association of sleep with the risk of incident liver cancer after further adjustment (N=356894)

| Sleep | HR (95% CI) |
| --- | --- |
| Unhealthy sleep (sleep score: 0~3) | **1.47 (1.16, 1.87)** |
| Individual component |  |
| Unfavorable sleep duration | **1.28 (1.002, 1.63)** |
| Evening chronotype | 1.20 (0.87, 1.66) |
| Insomnia | **1.44 (1.12, 1.85)** |
| Snoring | **1.31 (1.02, 1.69)** |
| Excessive daytime sleepiness | 1.31 (0.75, 2.29) |

Abbreviations: HR, hazard ratio; CI, confidence interval.

Models were further adjusted for liver function biomarkers (high: ≥median concentration/low: <median concentration) in addition to age, sex, ethnic, BMI, smoking status, alcohol consumption, healthy diet, physical activity, any treatment/medication taken, Townsend deprivation index, family history of cancer, and CRP.

**Table S9.** Associations of liver function biomarkers with the risk for incident liver cancer after further adjustment (N=356894).

| Liver function biomarkers | HR (95% CI) for per 10-unit increase | HR (95% CI) according to liver function biomarkers concentrations in quartiles | | | | *P* trend |
| --- | --- | --- | --- | --- | --- | --- |
|  |  | Q1 | Q2 | Q3 | Q4 |  |
| ALT (U/L) | **1.17 (1.15, 1.20)** | 1 (reference) | 0.74 (0.45, 1.21) | 1.26 (0.82, 1.94) | **3.25 (2.18, 4.84)** | **<0.001** |
| AST (U/L) | **1.20 (1.18, 1.22)** | 1 (reference) | 0.67 (0.40, 1.10) | 0.99 (0.63, 1.55) | **3.55 (2.45, 5.15)** | **<0.001** |
| TBIL (μmol//L) | **1.69 (1.47, 1.93)** | 1 (reference) | 0.93 (0.64, 1.37) | 1.03 (0.71, 1.50) | **1.68 (1.18, 2.38)** | **<0.001** |
| GGT (U/L) | **1.06 (1.06, 1.07)** | 1 (reference) | 0.95 (0.54, 1.67) | 1.33 (0.79, 2.26) | **5.17 (3.22, 8.32)** | **<0.001** |
| ALP (U/L) | **1.08 (1.07, 1.09)** | 1 (reference) | 0.87 (0.58, 1.31) | 1.14 (0.78, 1.67) | **2.12 (1.49, 3.00)** | **<0.001** |
| TP (g/L) | **1.81 (1.37, 2.39)** | 1 (reference) | 1.36 (0.94, 1.97) | 1.35 (0.93, 1.96) | **2.13 (1.51, 3.01)** | **0.001** |
| ALB (g/L) | **0.29 (0.18, 0.46)** | 1 (reference) | 0.79 (0.58, 1.07) | **0.54 (0.38, 0.77)** | **0.66 (0.47, 0.94)** | **<0.001** |

Abbreviations: HR, hazard ratio; CI, confidence interval; ALT, alanine transaminase; AST, aspartate transaminase; TBIL, total bilirubin; GGT, gamma-glutamyl transferase; ALP, alkaline phosphatase; TP, total protein; ALB, albumin.

Models were further adjusted for sleep score (healthy/unhealthy) and other liver function biomarkers (high: ≥median concentration/low: <median concentration) in addition to age, sex, ethnic, BMI, smoking status, alcohol consumption, healthy diet, physical activity, any treatment/medication taken, Townsend deprivation index, family history of cancer, and CRP.

*P* trend was tested by including the quartile order of liver function biomarkers as a continuous variable in the model.

**Table S10.** Association of sleep with the risk of incident liver cancer by inverse probability weighting analysis (N=356,894)

| Sleep | Model 1 | Model 2 | Model 3 |
| --- | --- | --- | --- |
|  | HR (95% CI) | HR (95% CI) | HR (95% CI) |
| Unhealthy sleep (sleep score: 0~3) | **1.48 (1.16, 1.88)** | **1.46 (1.15, 1.86)** | **1.46 (1.15, 1.86)** |
| Individual component |  |  |  |
| Unfavorable sleep duration | **1.58 (1.23, 2.02)** | **1.34 (1.04, 1.73)** | **1.33 (1.04, 1.72)** |
| Evening chronotype | 0.89 (0.64, 1.23) | 1.14 (0.82, 1.57) | 1.11 (0.80, 1.54) |
| Insomnia | **1.62 (1.26, 2.08)** | **1.43 (1.11, 1.84)** | **1.42 (1.10, 1.83)** |
| Snoring | 0.97 (0.76, 1.25) | 1.30 (1.00, 1.69) | **1.31 (1.01, 1.70)** |
| Excessive daytime sleepiness | **1.88 (1.07, 3.30)** | 1.30 (0.74, 2.27) | 1.26 (0.72, 2.21) |

Abbreviations: HR, hazard ratio; CI, confidence interval.

Model 1 was a crude model; Model 2 was adjusted for age, sex, ethnic, BMI, smoking status, alcohol consumption, healthy diet, and physical activity; Model 3 was adjusted for age, sex, ethnic, BMI, smoking status, alcohol consumption, healthy diet, physical activity, any treatment/medication taken, Townsend deprivation index, family history of cancer, and CRP.

**Table S11.** Associations of liver function biomarkers with the risk for incident liver cancer by inverse probability weighting analysis (N=356,894)

| Liver function biomarkers | HR (95% CI) for per 10-unit increase | HR (95% CI) according to liver function biomarkers concentrations in quartiles | | | | *P* trend |
| --- | --- | --- | --- | --- | --- | --- |
|  |  | Q1 | Q2 | Q3 | Q4 |  |
| ALT (U/L) | **1.18 (1.15, 1.21)** | 1 (reference) | 0.72 (0.43, 1.19) | 1.31 (0.84, 2.03) | **3.41 (2.28, 5.10)** | **<0.001** |
| AST (U/L) | **1.20 (1.18, 1.23)** | 1 (reference) | 0.62 (0.37, 1.04) | 0.97 (0.61, 1.55) | **3.71 (2.55, 5.40)** | **<0.001** |
| TBIL (μmol//L) | **1.65 (1.48, 1.84)** | 1 (reference) | 0.94 (0.64, 1.40) | 1.12 (0.75, 1.65) | **1.72 (1.19, 2.49)** | **0.001** |
| GGT (U/L) | **1.06 (1.06, 1.07)** | 1 (reference) | 0.91 (0.51, 1.63) | 1.26 (0.74, 2.15) | **4.91 (3.08, 7.83)** | **<0.001** |
| ALP (U/L) | **1.08 (1.07, 1.09)** | 1 (reference) | 0.76 (0.50, 1.18) | 1.09 (0.73, 1.63) | **1.97 (1.37, 2.83)** | **<0.001** |
| TP (g/L) | **1.76 (1.31, 2.36)** | 1 (reference) | 1.34 (0.91, 1.96) | 1.41 (0.96, 2.09) | **2.14 (1.50, 3.05)** | **<0.001** |
| ALB (g/L) | **0.28 (0.16, 0.48)** | 1 (reference) | **0.72 (0.53, 0.995)** | **0.56 (0.39, 0.80)** | **0.61 (0.42, 0.87)** | **0.002** |

Abbreviations: HR, hazard ratio; CI, confidence interval; ALT, alanine transaminase; AST, aspartate transaminase; TBIL, total bilirubin; GGT, gamma-glutamyl transferase; ALP, alkaline phosphatase; TP, total protein; ALB, albumin.

Models were adjusted for age, sex, ethnic, BMI, smoking status, alcohol consumption, healthy diet, physical activity, any treatment/medication taken, Townsend deprivation index, family history of cancer, and CRP.

*P* trend was tested by including the quartile order of liver function biomarkers as a continuous variable in the model.

**Table S12.** Association of sleep with the risk of incident liver cancer in mortality competing risk model (N=356,894)

| Sleep | HR (95% CI) | |
| --- | --- | --- |
|  | Overall | ≥60 years |
| Unhealthy sleep (sleep score: 0~3) | **1.44 (1.12, 1.84)** | **1.43 (1.05, 1.96)** |
| Individual component |  |  |
| Unfavorable sleep duration | **1.28 (1.00, 1.64)** | 1.16 (0.84, 1.59) |
| Evening chronotype | 1.16 (0.84, 1.61) | 1.28 (0.83, 1.97) |
| Insomnia | **1.42 (1.10, 1.83)** | **1.44 (1.05, 1.98)** |
| Snoring | 1.28 (0.99, 1.65) | 1.27 (0.92, 1.75) |
| Dozing | 1.27 (0.72, 2.21) | 1.45 (0.76, 2.74) |

Abbreviations: HR, hazard ratio; CI, confidence interval.

The number of deaths without having been diagnosed with liver cancer was 23,072 and 16,358 in the total population and in the population ≥60 years, respectively.

Models were adjusted for age, sex, ethnic, BMI, smoking status, alcohol consumption, healthy diet, physical activity, any treatment/medication taken, Townsend deprivation index, family history of cancer, and CRP.

**Table S13.** Associations of liver function biomarkers with the risk for incident liver cancer in mortality competing risk model (N=356,894)

| Liver function biomarkers | HR (95% CI) for per 10-unit increase | |
| --- | --- | --- |
|  | Overall | ≥60 years |
| ALT (U/L) | **1.17 (1.15, 1.20)** | **1.18 (1.15, 1.20)** |
| AST (U/L) | **1.20 (1.17, 1.22)** | **1.21 (1.18, 1.24)** |
| TB (μmol//L) | **1.54 (1.37, 1.73)** | **1.40 (1.22, 1.62)** |
| GGT (U/L) | **1.06 (1.05, 1.06)** | **1.06 (1.05, 1.06)** |
| ALP (U/L) | **1.08 (1.07, 1.09)** | **1.07 (1.05, 1.08)** |
| TP (g/L) | **1.78 (1.33, 2.38)** | **1.86 (1.30, 2.65)** |
| ALB (g/L) | **0.31 (0.18, 0.53)** | **0.40 (0.21, 0.75)** |

Abbreviations: HR, hazard ratio; CI, confidence interval; ALT, alanine transaminase; AST, aspartate transaminase; TBIL, total bilirubin; GGT, gamma-glutamyl transferase; ALP, alkaline phosphatase; TP, total protein; ALB, albumin.

The number of deaths without having been diagnosed with liver cancer was 23,072 and 16,358 in the total population and in the population ≥60 years, respectively.

Models were adjusted for age, sex, ethnic, BMI, smoking status, alcohol consumption, healthy diet, physical activity, any treatment/medication taken, Townsend deprivation index, family history of cancer, and CRP.


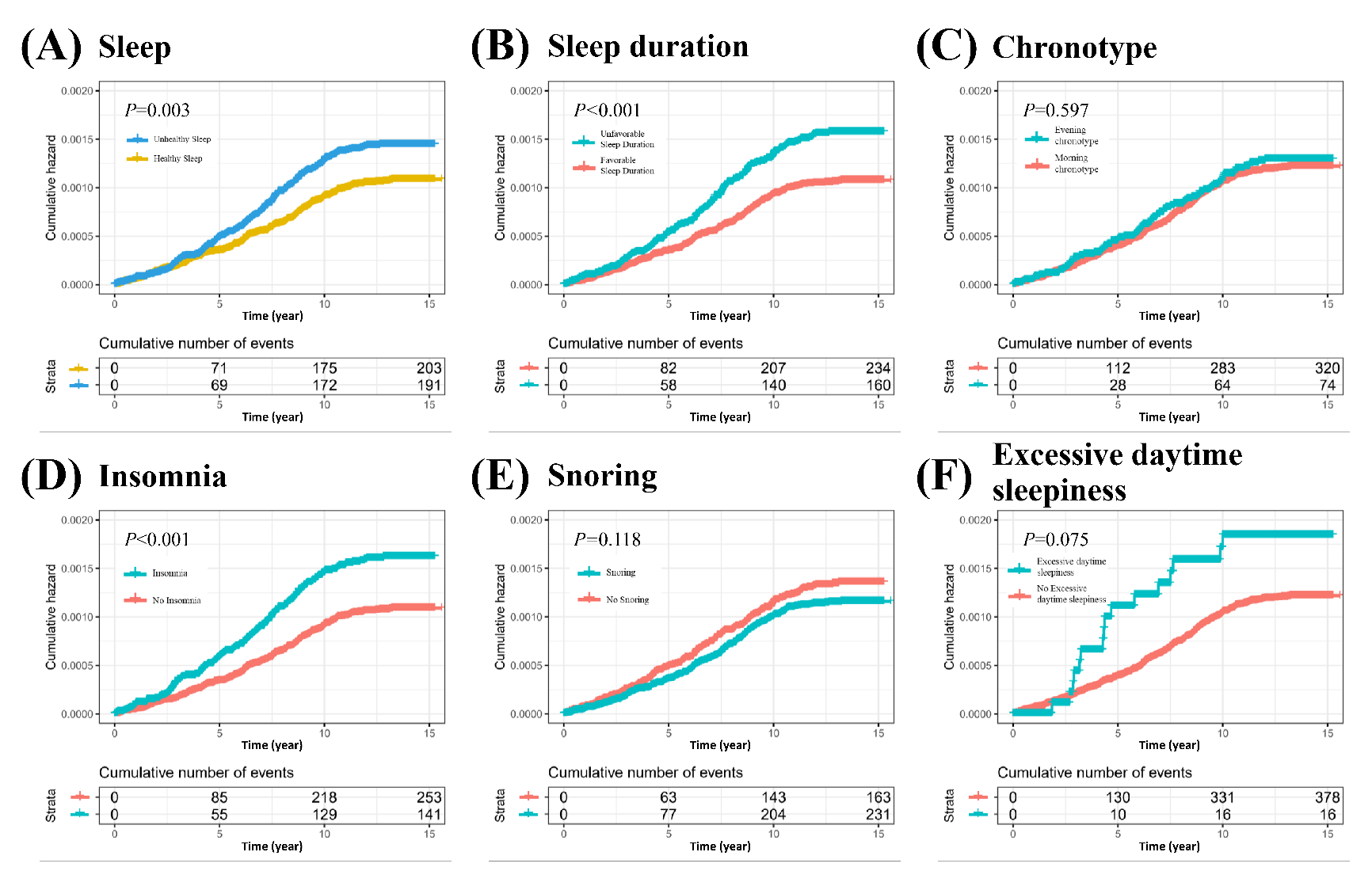


**Fig. S1.** Kaplan-Meier curves of sleep and independent sleep traits with incident liver cancer (N=356,894)

**
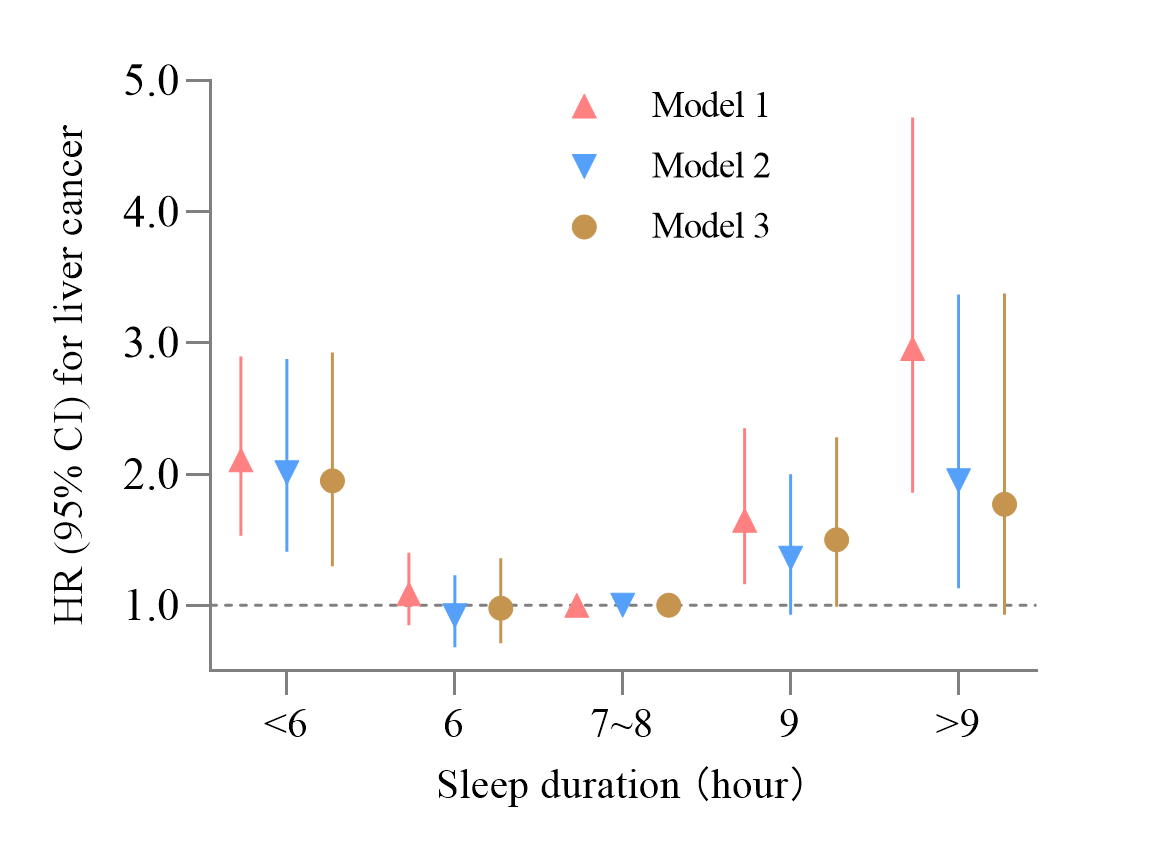
**

**Fig. S2.** Association of sleep duration with the risk of incident liver cancer across different hours (N=356,894)

Abbreviations: HR, hazard ratio; CI, confidence interval.

HR (95% CI) across different sleep durations were presented with favorable sleep duration (7~8 hours) serving as a reference group.

Model 1 was a crude model; Model 2 was adjusted for age, sex, ethnic, BMI, smoking status, alcohol consumption, healthy diet, and physical activity; Model 3 was adjusted for age, sex, ethnic, BMI, smoking status, alcohol consumption, healthy diet, physical activity, any treatment/medication taken, Townsend deprivation index, family history of cancer, and CRP.

**
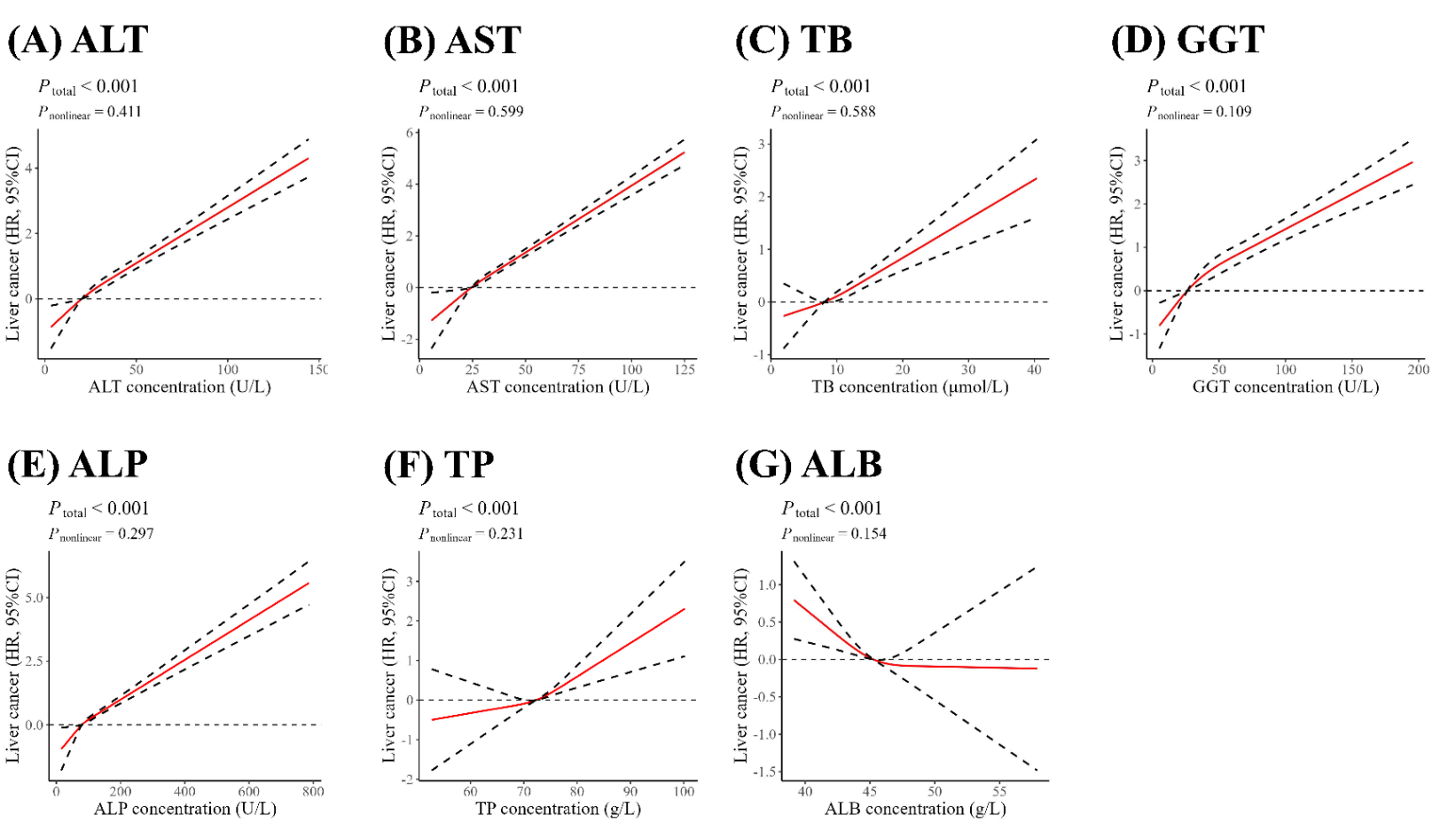
**

**Fig. S3.** Associations between liver function biomarkers and the risk of incident liver cancer by restricted cubic spline regression (N=356,894)

Curves were delineated by restricted cubic splines with 3 knots at 10^th^, 50^th^ and 90^th^ percentiles.

Abbreviations: HR, hazard ratio; CI, confidence interval; ALT, alanine transaminase; AST, aspartate transaminase; TBIL, total bilirubin; GGT, gamma-glutamyl transferase; ALP, alkaline phosphatase; TP, total protein; ALB, albumin.

Models were adjusted for age, sex, ethnic, BMI, smoking status, alcohol consumption, healthy diet, physical activity, any treatment/medication taken, Townsend deprivation index, family history of cancer, and CRP.
